# Targeting of X-linked inhibitor of apoptosis protein and PI3-kinase/AKT signaling by embelin suppresses growth of leukemic cells

**DOI:** 10.1371/journal.pone.0180895

**Published:** 2017-07-13

**Authors:** Kirti S. Prabhu, Kodappully S. Siveen, Shilpa Kuttikrishnan, Ahmad Iskandarani, Magdalini Tsakou, Iman W. Achkar, Lubna Therachiyil, Roopesh Krishnankutty, Aijaz Parray, Michal Kulinski, Maysaloun Merhi, Said Dermime, Ramzi M. Mohammad, Shahab Uddin

**Affiliations:** 1 Translational Research Institute, Academic Health System, Hamad Medical Corporation, Doha, State of Qatar; 2 Translational Cancer Research Facility, National Center for Cancer Care and Research (NCCCR), Hamad Medical Corporation, Doha, State of Qatar; Universite du Quebec a Trois-Rivieres, CANADA

## Abstract

The X-linked inhibitor of apoptosis (XIAP) is a viable molecular target for anticancer drugs that overcome apoptosis-resistance of malignant cells. XIAP is an inhibitor of apoptosis, mediating through its association with BIR3 domain of caspase 9. Embelin, a quinone derivative isolated from the *Embelia ribes* plant, has been shown to exhibit chemopreventive, anti-inflammatory, and apoptotic activities via inhibiting XIAP activity. In this study, we found that embelin causes a dose-dependent suppression of proliferation in leukemic cell lines K562 and U937. Embelin mediated inhibition of proliferation correlates with induction of apoptosis. Furthermore, embelin treatment causes loss of mitochondrial membrane potential and release of cytochrome c, resulting in subsequent activation of caspase-3 followed by polyadenosin-5’-diphosphate-ribose polymerase (PARP) cleavage. In addition, embelin treatment of leukemic cells results in a decrease of constitutive phosphorylations/activation level of AKT and downregulation of XIAP. Gene silencing of XIAP and AKT expression showed a link between XIAP expression and activated AKT in leukemic cells. Interestingly, targeting of XIAP and PI3-kinase/AKT signaling augmented inhibition of proliferation and induction of apoptosis in leukemic cells. Altogether these findings raise the possibility that embelin alone or in combination with inhibitors of PI3-kinase/AKT pathway may have therapeutic usage in leukemia and possibly other malignancies with up-regulated XIAP pathway.

## Introduction

Resistance to apoptosis is one of the hallmarks that promotes cancer development and progression in various cancers including leukemia [1, 2]. Furthermore, escape from apoptosis is the important causes of failure of antileukemic effects of many conventional therapeutic drugs as many of anticancer drugs exhibit anticancer activity via inducing apoptosis in malignant cells [3]. X-linked inhibitor of apoptosis protein (XIAP) is a prominent protein member of the inhibitor of apoptosis (IAP) that collectively involved inhibition of apoptosis and thereby improving the survival of cancer cell [4–6]. XIAP is the only member of the IAPs that has been shown to inhibit the functionality of both; the initiation caspase (caspase-9) as well as executioner caspase (caspase-3) thereby limiting the role of apoptosis in cancer cells [7, 8]. There is accumulating evidence that XIAP is involved in regulating apoptosis sensitivity of malignant cells and also exhibits prognostic implications [9, 10] as high expression of XIAP has been reported in leukemic blasts and correlates with poor survival [11]. XIAP protein and mRNA levels have been associated with chemoresistance and poor clinical outcome in leukemic patients [11–13]. Overexpression of XIAP has been shown to be associated with activated AKT in many cancers including leukemia [14, 15]. Activation of AKT is involved in the protection of XIAP degradation by chemotherapeutic agents in malignant cells [16]. Recently we and other have shown a functional association of AKT and XIAP in cancer cells [4, 17, 18].

Embelin (2, 5-dihydroxy-3-undecyl-1, 4- benzoquinone) is a natural benzoquinone isolated from the fruit of the *Embelia ribes* [19]. Embelin exhibits anti-cancer and anti-inflammatory activity in various cancer cells [4, 5, 20]. Embelin is a potent small molecule inhibitor of XIAP which prevents the binding of XIAP to procaspase-9 [19] and exhibits cytotoxic effects via suppressing the activity various signaling cascades including PI3-kinase/AKT in a variety of cancer cell lines [4, 5, 21–23]. Embelin has been found to sensitise acute myeloid leukemic cells to TRAIL through the inhibition of XIAP and inactivation of NF-kB activity [24–26]. Therefore, we investigated the antitumor activity of embelin using leukemic cell lines, with an interest in supporting previous findings that XIAP can be considered as potential target for anticancer therapy [27, 28]. Our data showed that embelin treatment of leukemic cells inhibited cell proliferation via inducing apoptosis. Embelin treatment suppresses constitutively activated AKT and downregulates XIAP expression resulting in mitochondrial-caspase mediated apoptosis. Interestingly, co-treatment of leukemic cells with LY294002 and embelin augmented apoptotic cell death.

## Materials and methods

### Reagents and antibodies

Embelin was purchased from Tocris Bioscience (Minneapolis, MN). zVAD-fmk was purchased from Calbiochem (San Diego, CA). Antibodies against caspase-9, caspase-8, Bid, Bcl-xL, phospho AKT and cleaved caspase-3, caspase-3 were purchased from Cell Signaling Technologies (Beverly, MA). Cytochrome *c*, PARP and GAPDH antibodies were purchased from Santa Cruz Biotechnology, Inc. (Santa Cruz, CA, USA). XIAP antibody was purchased from Abcam (Cambridge, England). Annexin V-FITC, Propidium iodide staining solution, Hoechst 33342 Solution, BD Cytofix/Cytoperm plus fixation and permeabilization solution kit, PE Active Caspase-3 Apoptosis Kit and Apoptosis, BD MitoScreen (JC-1) Kit, DNA Damage and Cell Proliferation Kit were purchased from BD Biosciences (NJ, USA). AKT, XIAP and Scrambled control siRNA were purchased from Qiagen (Valencia, CA, USA). Lipofectamine 2000 was purchased from Invitrogen (Carlsbad, CA). 5-(2-Benzothiazolyl)-3-ethyl-2-[2-(methylphenylamino)ethenyl]-1-phenyl-1H-benzimidazolium iodide was purchased from Sigma Aldrich (USA).

### Cell culture

K562 and U937 cells were grown in RPMI 1640 medium containing 10% (v/v) fetal bovine serum and appropriate antibiotics at 37 ^0^C in a humidified incubator with 5% CO_2_.

### 3-(4, 5-Dimethylthiazol-2-yl)-2, 5-diphenyltetrazolium bromide assays

MTT assay was performed as described previously [29]. Briefly, 1x10^4^ cells were added in 96 cell culture and treated with various doses of embelin in a final volume of 0.2 mL for 24 h. At the end of incubation, 20 μl (5 mg/mL) of MTT reagent was added to each well. After 3–4 h incubation at 37 ^0^C, 100 μL 20% SDS was added to each well and incubation was continued. Optical density was at 570 nm. Percent cell viability was calculated as OD of the experiment samples/OD of the control × 100.

### Cell cycle analysis

Cell cycle analysis was determined as described previously [30]. Briefly, leukemic cell lines were treated with indicated concentrations of embelin for 24 h. Following the drug treatment, cells were incubated with Hoechst 33342 (8 μg/mL) in complete medium for 30 min 37°C. The cells were then washed twice with PBS and analyzed by flow cytometry using a BD LSRFortessa analyzer (BD Biosciences, USA).

### AnnexinV/propidium iodide dual staining

K562 and U937 cell lines were treated with different concentrations of embelin for 24 h, and then harvested and washed with PBS. After staining with fluorescein-conjugated annexin-V and propidium iodide in 1x annexin binding buffer for 20 min. the cell were analyzed by flow cytometry to quantify the live (Annexin-FITC^-ive^, PI^-ive^), early (Annexin-FITC^+ive^, PI^-ive^) and late (Annexin-FITC^+ive^, PI^+ive^) apoptotic cells and necrosis (Annexin-FITC^-ive^, PI^+ive^), as described previously [31]. Percentage of cells in early and late apoptosis were combined and expressed as percentage apoptosis.

### Gene silencing study using siRNA technology

Transfections of AKT, XIAP and Scrambled siRNA were performed using Lipofectamine 2000 according to the manufacturer’s instructions. After 6 h of incubation, lipid and siRNA complex was removed from cells and fresh growth medium was added and incubated for 48 h. Cells were lysed and immunoblotted with XIAP and p-AKT antibodies as described in the figure legends.

### Cell lysis and immunoblotting

Embelin treated K562 and U937 cells were lysed with laemmeli buffer. Quantification of proteins were performed using the ND-1000 (Nanodrop Technologies, Thermoscientific, USA). After adding β-mercaptoethanol, 25–50 μg of protein samples were resolved by SDS-PAGE and transferred to polyvinylidene difluoride (PVDF) membrane (Immobilon, Millipore, Billerica, MA). Immunoblotting was performed using various antibodies as described previously [32]. The blots were developed and further visualized under a chemidoc system (Amersham, Bio-Rad, USA).

### Assay for measurement of DNA double strand breaks

Leukemic cells after treatment with various doses of embelin were fixed and permeabilized using BD Cytofix/Cytoperm Plus Fixation and Permeabilization Solution Kit. 1 x 10^5^ cells were stained with 5 μL H2AX (pS139)-Alexa Fluor 647 antibody and then measured by Flow Cytometry, as described earlier, to quantify the DNA double strand breaks [33].

### Measurement of Membrane Potential Measurement (MMP)

JC1 stain kit was used to determine the MMP in K562 and U937 cells treated with embelin, as described previously [34]. Briefly, cells were stained with JC-1 stain in dark according to the protocol of manufacturer. MMP in each samples were determined by Flow Cytometry, which is denoted by a reduction in red flouresence.

### Measurement of active Caspase-3 and cleaved PARP

Leukemic cells were treated with embelin, as described in the legends. BD Cytofix/Cytoperm Plus Fixation kit was used to fix and permeabilize embelin treated and untreated cells according to the manufacturer’s protocol. 1 x 10^5^ cells were stained with 5 μL anti- Active Caspase-3-BV605 and 5 μL PARP Cleaved Form-AF700 antibodies for 30 min. The cells were washed and analyzed by Flow Cytometry, as described earlier[33].

### Cytochrome *C* release assay

K562 and U937 cells were treated with 10, 25–50 μM embelin for 24 h, cells were harvested and resuspended in hypotonic buffer. Mitochondrial and cytosolic fraction was isolated as described earlier [35]. Protein from cytosolic and mitochondrial fractions of each sample were analyzed by immunoblotting using an Anti-cytochrome *c* and tubulin antibody.

## Statistical analysis

Comparisons between groups were made using the paired Student’s t-test. Values of P<0.05 were considered statistically significant. Statistical signifiacne is shown with * (P<0.001), # (P<0.01) and $ (P<0.05). Data are expressed as the mean ± S.D.

## Results

### Treatment of leukemic cells with embelin causes suppression ofgrowth and induces apoptosis

**Fig 1:**
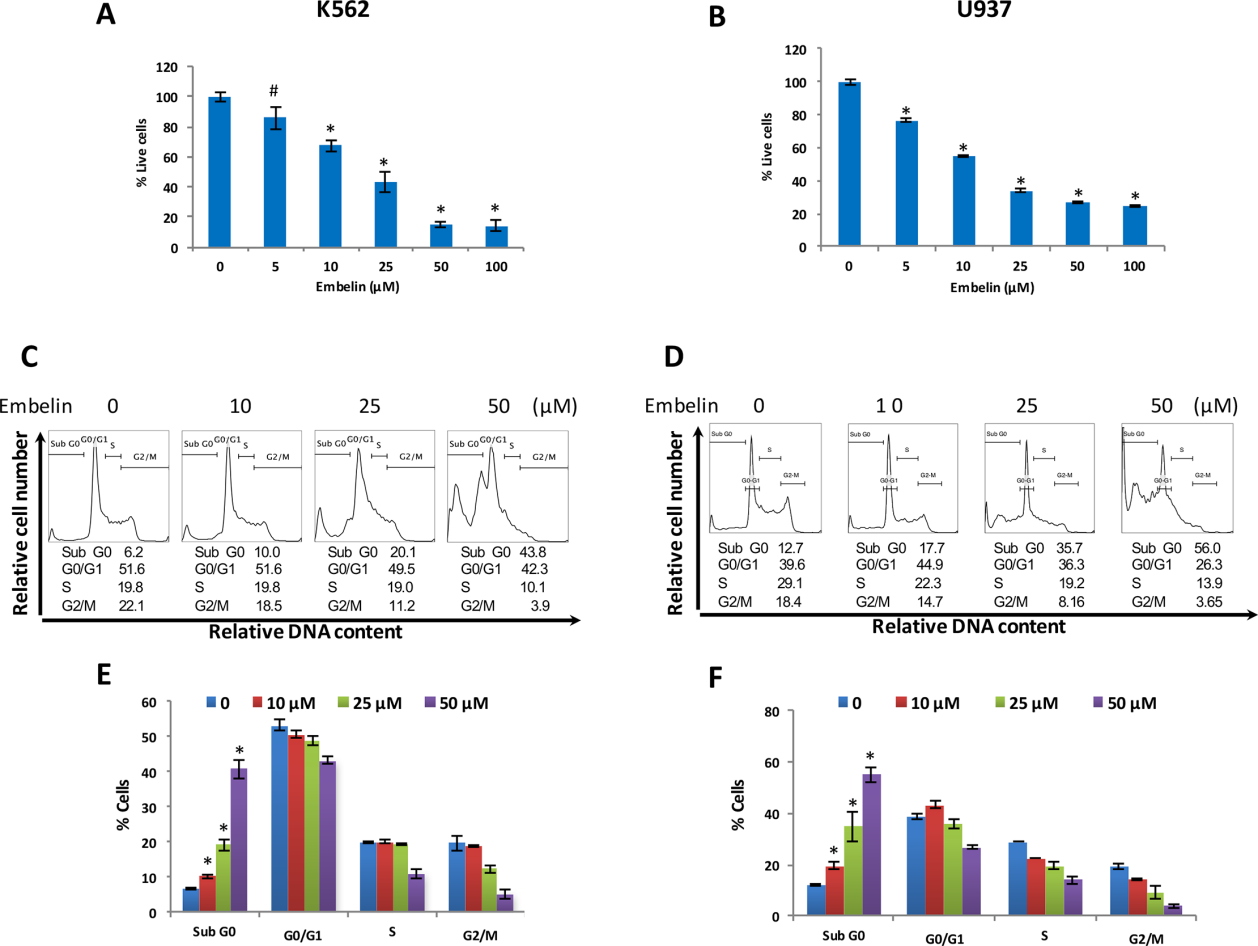
Effects of embelin on proliferation and cell cycle progression in leukemic cells. Embelin inhibits the cell viability of leukemic cells. K562 **(A)** and U937 **(B)** cells were incubated with embelin as indicated in the figure for 24 h. Cell proliferation assays were performed using MTT as described in Materials and Methods. The graph displays the mean +/- SD (standard deviation) of three independent experiments. *P<0.001 Cell cycle fractions of embelin treated leukemic cell lines. K562 **(C)** and U937 **(D)** cells were treated with embelin as indicated in figure for 24 h. Thereafter, the cells were stained with Hoechst 33342, and analyzed for DNA content by flow cytometry as described in Materials and Methods. A representative of three independent experiments is depicted in the figure. Embelin significantly increased subG0 population in K562 **(E)** and U937 **(F)** cells. The graph displays the mean +/-SD of three independent of experiments *P < 0.001.

**Fig 2:**
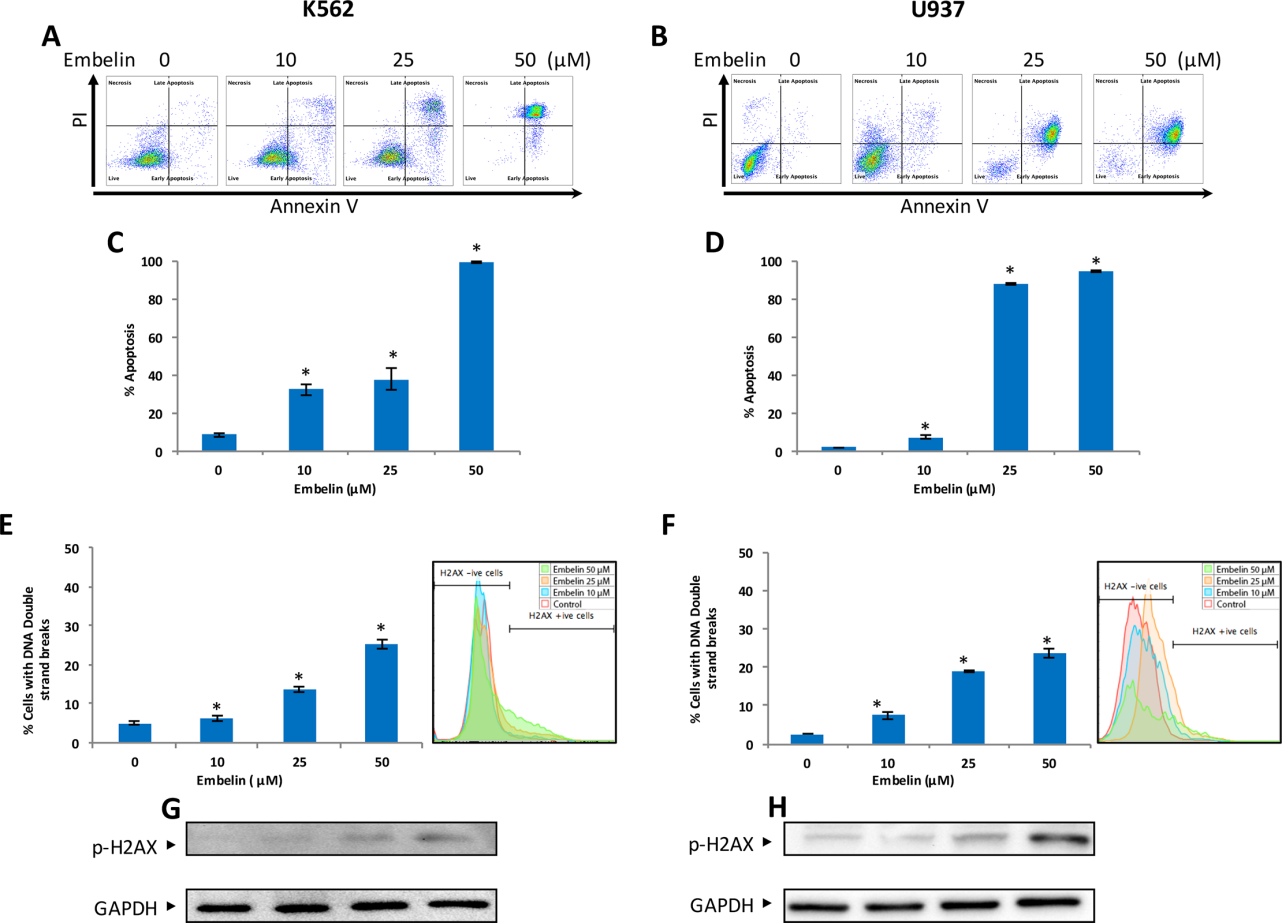
Embelin-induced apoptosis in leukemic cell lines. K562 **(A)** and U937 **(B)** cells were treated with embelin as indicated in figure for 24 h. Cells were then, subsequently stained with fluorescein-conjugated annexin-V and propidium iodide (PI) and analyzed by flow cytometry. A representative of three independent experiments is depicted in the figure. Embelin-induced dose-dependent apoptosis in leukemic cells. K562 **(C)** and U937 **(D)** cell line were treated with embelin and apoptosis was measued by flow cytometry after staining with annexin-V and PI. Percentage of apoptosis relative to untreated cells was calculated as described in Material and Methods. The graph displays the mean +/-SD of three independent of experiments. Embelin-induced DNA double strand breaks in leukemic cells. K562 **(E)** and U937 **(F)** cells were treated with embelin as indicated in the figure for 24 h. Quantification of DNA double strand breaks were assayed as described in Material and Method section. The graph (left panel) displays the mean +/-SD of three independent of experiments *P < 0.001. The right panel shows an overlay of H2AX (pS139)-Alexa Fluor 647 fluoresence intensity of different treatment conditions Detection of p-H2AX in embelin treated leukemic cell lines. K562 **(G)** and U937 **(H)** cells were treated with embelin as indicated in figure for 24 h and cells were subsequently lysed and immunoblotted with anti-p H2AX antibody and GAPDH for equal loading.

Initially, we determined whether treatment of leukemic cells with embelin could cause inhibition of cell proliferation. To achieve this objective, K562 and U937 cells were treated with indicated doses of embelin for 24 h and proliferation was determined by MTT assays. As shown in Fig 1A and 1B, embelin treatment of K562 and U937 cells prevented cell proliferation significantly at the dose 5μM and above in a dose-dependent manner (IC50 was found in a range of 12–18μM). In the next series of experiments, we determined cell cycle analysis and apoptosis using various methodologies. As shown in Fig 1C–1F, embelin treatment resulted in an increase in the subG0/Apoptotic fraction from 6.2% in untreated cells to 10.0, 20.1 and 43.8% in K562 treated with 10, 25 and 50 μM embelin respectively. Similarly in U937 cells, 12.7% in untreated to 17.7, 35.7 and 56% respectively in embelin treated cells. This increase in SubG0 in response to embelin suggests that decrease in cell viability was due to induction of apoptosis. We performed a number of apoptotic cell death assays including annexinV/PI dual staining and DNA double strand breaks following embelin treatment to further confirm the apoptotic process. Using annexinV/PI dual staining embelin treatment of K562 cells with 10, 25 and 50 μM induced 33.0, 38.0 and 99.0% apoptosis. Similarly, 10, 20 and 50 μM embelin resulted in 7.0, 88.0 and 95.0% apoptosis respectively in U937 cell line (Fig 2A–2D).

Phosphorylation of H2AX at Serine 139 has been shown to correlate with induction of double stranded breaks [35] and subsequently, is a marker for DNA damage [36]. Quantification of phospho-γ-H2AX (S139) by flow cytometry and western blot was performed in K562 and U937 cells treated with embelin. Our results show that embelin treatment induced a significant dose-dependent DNA double strand breaks (Fig 2E–2H).

### Embelin activates the intrinsic apoptotic pathway in leukemic cells

**Fig 3:**
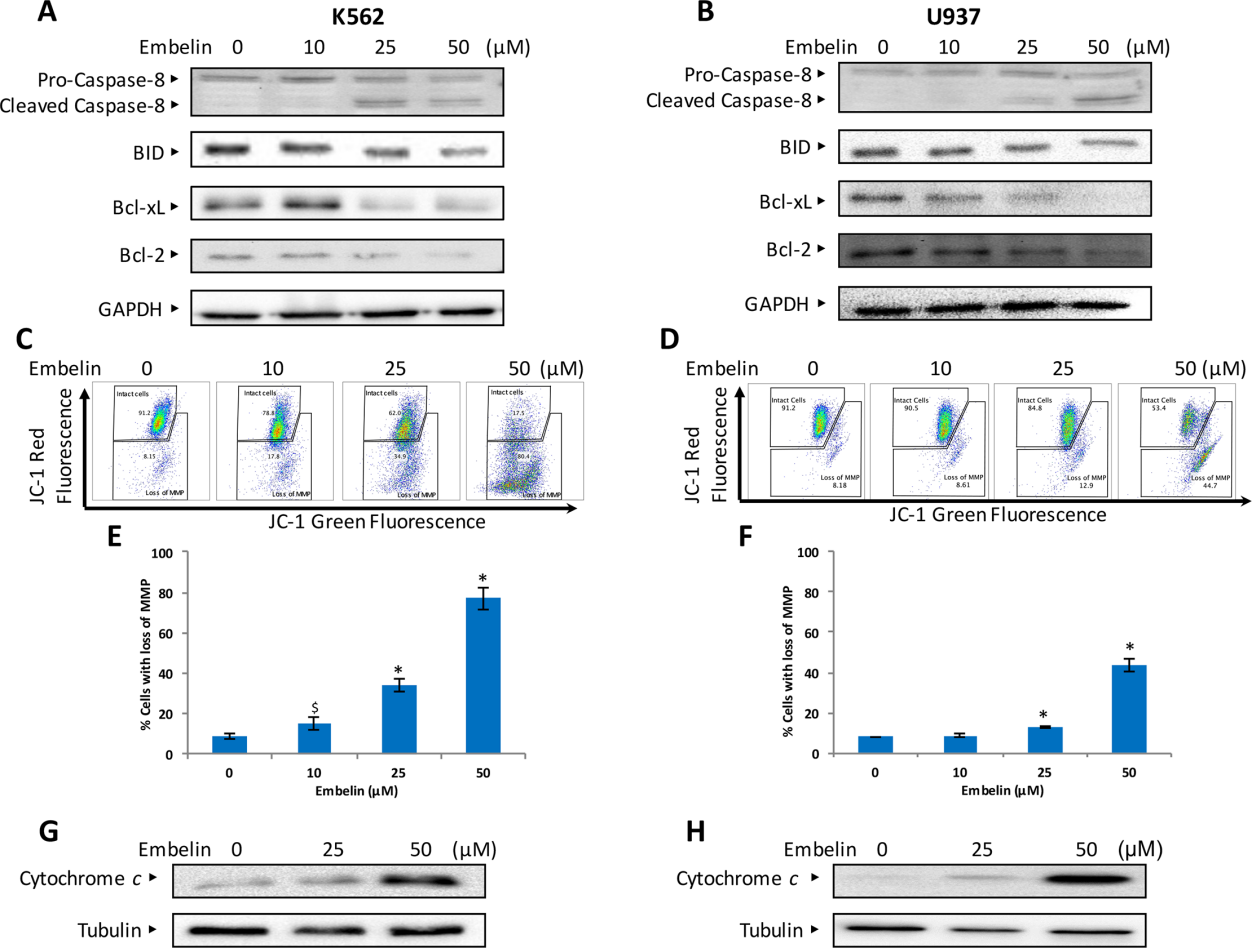
Embelin-induced activation of caspase 8 and mitochondrial apoptotic pathway. Embelin-induced activation of caspase 8 and Bid in leukemic cell lines. K562 **(A)** and U937 **(B)** cells were treated with embelin as indicated in figure for 24 h. Cells were lysed and immunoblotted with antibodies against caspase 8, Bid, Bcl-xl, Bcl2 and GAPDH. Embelin treatment causes the loss of MMP in leukemic cells. K562 **(C)** and U937 **(D)** cells were treated with embelin for 24 h. After JC1 staining cells were analyzed by flow cytometry as described in Materials and Methods. A representative of three independent experiments is depicted in the figure. The dose-dependent loss of MMP in K562 (E) and U937 **(F)** cells were quantified and represented as a bar graph. The graph displays the mean +/-SD of three independent of experiments. $*P* <0.05 and *P < 0.001. Embelin-induced release of cytochrome *c* in leukemic cells. K562 **(G)** and U937 **(H)** cells were treated with 25 and 50 μM of embelin for 24 h. Proteins from cytoplasmic fraction were immunoblotted with an antibody against cytochrome *c* and tubulin.

Although caspase-8 is involved in receptor mediated or extrinsic type of apoptotic pathway, it can also activate mitochondrial-mediated apoptotic pathway via truncation of Bid, a member of Bcl-2 family protein [37]. Truncated Bid has been shown to downregulate Bcl-2 and Bcl-xL expression [4, 5, 20]. Therefore, we determined whether embelin-mediated apoptosis involves activation of caspase-8 and Bid in mitochondrial or intrinsic apoptotic pathway. Embelin treatment of leukemic cells resulted in a reduction in full-length band caspase and an increase of cleaved caspase-8, indicating activation of caspase-8. In addition, embelin also led to a decrease in full-length Bid protein suggesting that embelin causes truncation of Bid. Truncated Bid has been shown to downregulate the expression of Bcl-2, Bcl-xL proteins and upregulate the expression of proapoptotic Bax protein (Fig 3A and 3B).

**Fig 4:**
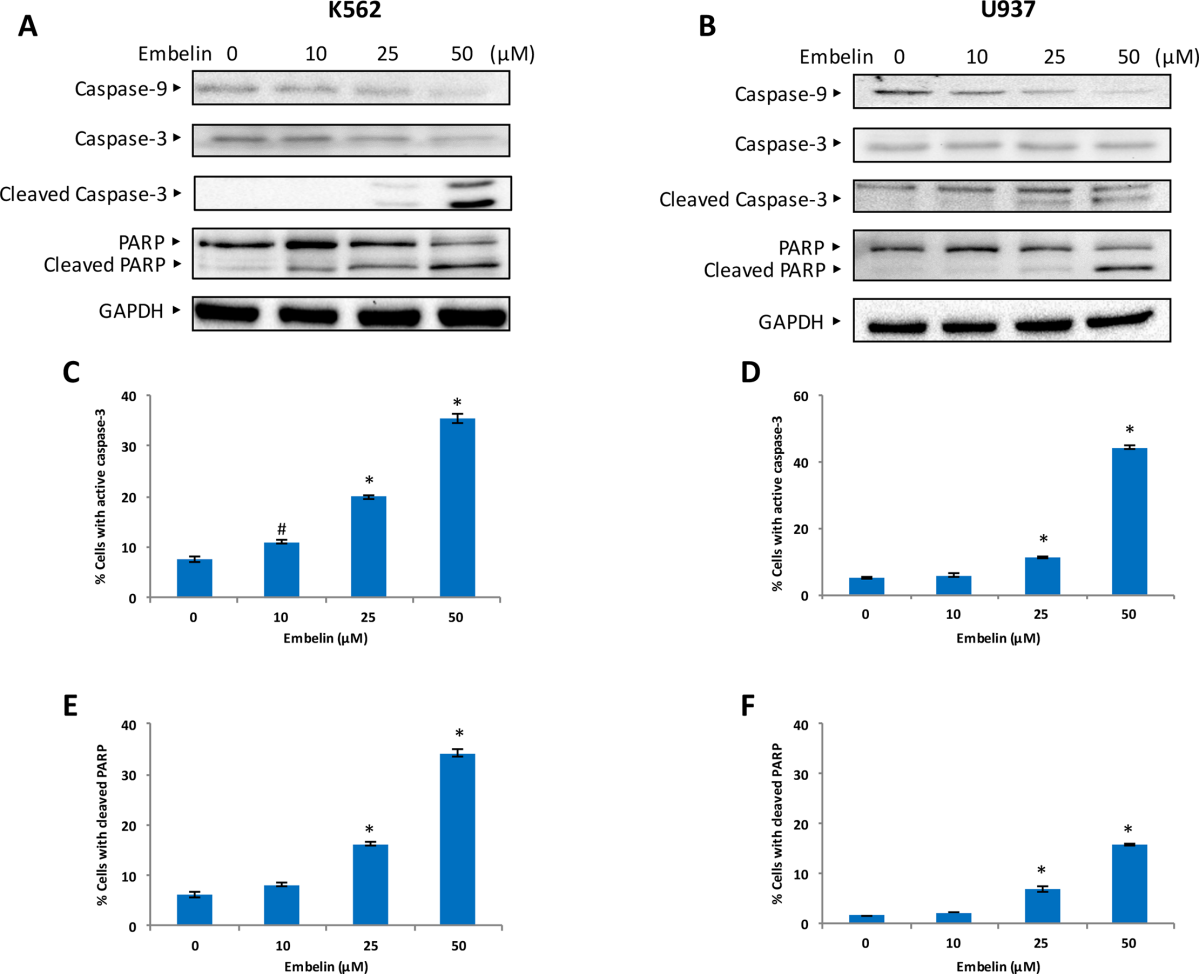
Embelin-induced activation of caspase-9, caspase-3, and cleavage of PARP in leukemic cell lines. Embelin-mediated activation of the caspase cascades in leukemic cells. K562 **(A)** and U937 **(B)** cells were treated with embelin as indicated in figure for 24 h. Cells were lysed and immunoblotted with antibodies against caspase-9, caspase-3, cleaved caspase-3-3, PARP, and GAPDH. Embelin–induced activation of caspases 3 in leukemic cells K562 **(C)** and U937 **(D)** cells when treated with embelin for 24 h. Subsequent cleavage of PARP was determined by flow cytometry as described in Materials and Methods. A representative of three independent experiments is depicted in the figure. Percentage of K562 **(E)** and U937 **(F)** cells for cleaved PARP. The graph displays the mean +/- SD of three independent of experiments $ *P*<0.05, # *P*<0.01 and * *P* < 0.001.

**Fig 5:**
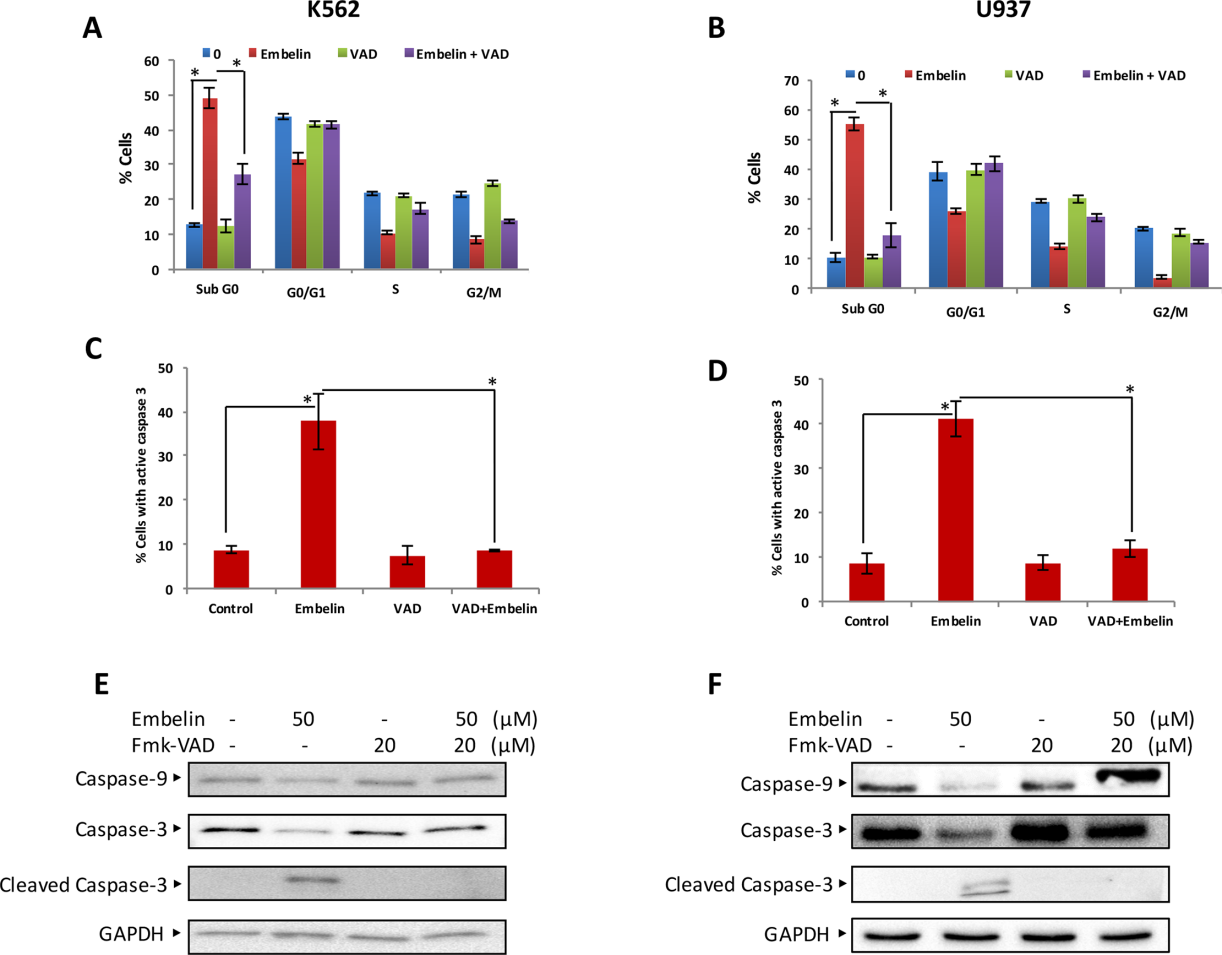
Effect of z-VAD-fmk on Embelin-induced apoptosis. K562 **(A)** and U937 **(B)** cells were pre-treated with 20 μM z-VAD/fmk for 2 h and subsequently treated with 50 μM embelin for 24 h. Cell cycle analysis was determined as described in Material and Methods. A representative of three independent experiments is depicted in the figure. Percentage of cells in G0/G1, S and G2/M phases of cell cycle as well as SubG0 cells were calculated for K562 (A) and U937 (B). The graph displays the mean +/- SD of three experiments. z-VAD/fmk abrogates embelin-induced caspase-3 activation. K562 cells **(C)** and U937 **(D)** when pre-treated with 20 μM z-VAD/fmk for 2 h and subsequently treated with 50 μM embelin for 24 h. Caspase-3 activation was determined by flow cytometry as described in Material and Methods. The graph displays the mean +/- SD (standard deviation) of three experiments. z-VAD/fmk prevented embelin-induced activation of caspases. K562 **(E)** and U937 **(F)** cells were pre-treated with 20 μM z-VAD/fmk for 2 h and subsequently treated with 50 μM embelin for 24 h and cells were lysed and 50 μg protein were immunoblotted with antibodies against caspase-9, pro-caspase-3, cleaved caspase-3, and GAPDH.

The Bcl-2 family member proteins, Bcl-2, Bcl-xL and Bax expression, have shown to be involved in the maintenance of the integrity of mitochondrial membrane and its membrane potential. The intact mitochondrial membrane also protects leakage of cytochrome *c*. We investigated whether embelin treatment of leukemic cell lines causes loss of MMP. K562 and U937 cells were treated with embelin and labeled using JC1, a dye used to measure MMP by flow cytometry. As shown in Fig 3C and 3F embelin causes a dose dependent significant loss in MMP in both cell lines. Embelin treatment causes membrane depolarization which leads to decrease in JC1 Red fluorescence intensity. As the loss of MMP mediates the release of cytochrome *c* to cytosol, we sought to determine whether embelin-treated cells shows the presence of cytochrome *c* in the cytosol. As expected, we found increase in cytosolic detection of cytochrome *c* in K562 and U937 cells treated with embelin in a dose-dependent manner (Fig 3G and 3H). These findings show that embelin-treatment of cells led to the formation of mitochondrial membrane transition pores resulting in loss of MMP and accumulation of cytochrome *c* in the cytosol. In the cytosol, a complex is formed in the presence of cytochrome *c*, caspase 9, Apaf-1 and ATP, known as the apoptosome [38]. Caspases are hallmark markers of apoptotic cell death [39]. As embelin-treatment causes accumulation of cytochrome *c*, we investigated whether caspases were activated in leukemic cells under this treatment condition. K562 and U937 cells were treated with embelin and immunoblotted with caspase-9, procaspase-3, cleaved caspase-3 and PARP. As shown in Fig 4A and 4B, embelin induces caspases in a dose-dependent manner. In addition, caspase activity was also assayed by a BD active caspase assay kit as described in Material and Methods. As shown in Fig 4C–4F there is a significant increase in the levels of active caspase-3 and cleaved PARP at 25 and 50 μM embelin in both cell lines. In addition, z-VAD-fmk, an inhibitor of caspases partially reversed embelin-induced apoptosis and activation of caspases in K562 and U937 cells indicating apoptosis mediated by embelin is not completely caspase dependent (Fig 5A–5F). Therefore, we next sought to demonstrate whether embelin induces autophagy in leukemic cells. K562 and U937 cells were treated with embelin in a dose-dependent manner. We observed increased expression of LC3 II (S1 Fig) in both K562 and U937 cells treated with embelin. Thus, confirming embelin induced concomitant cell death by apoptosis and autophagy.

### Functional relationship of AKT and XIAP in leukemic cells

**Fig 6:**
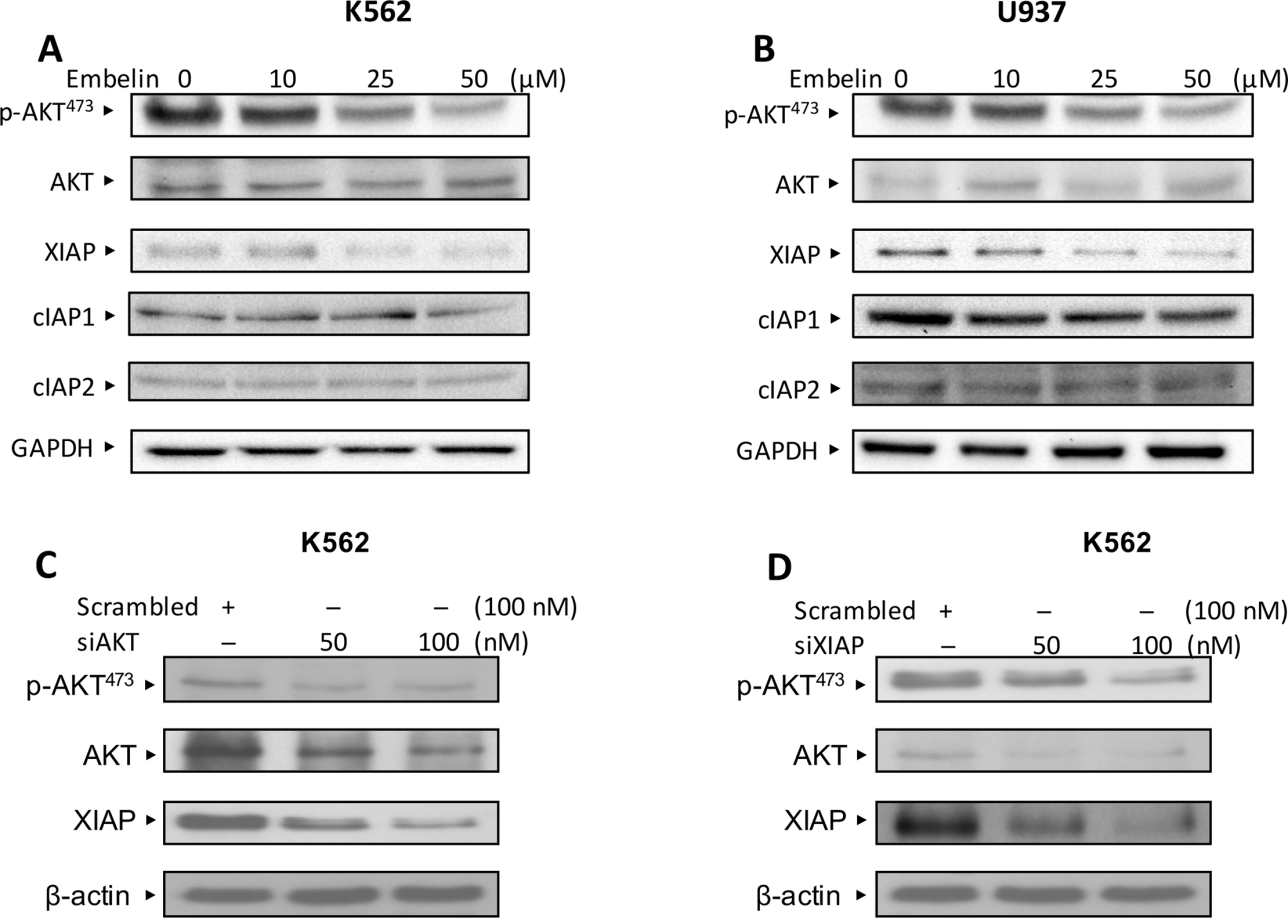
Embelin treatment inactivates AKT and down-regulates XIAP expression in leukemic cell lines. K562 **(A)** and U937 **(B)** cells were treated embelin as indicated in the figure. After 24 h of treatment, cells were lysed and proteins were immunoblotted with antibodies against p-Akt, XIAP, cIAP1, cIAP2 and GAPDH. **(C)** AKT knockdown using specific siRNA inactivates AKT and down-regulates XIAP expression in K562 cells. K562 cells were transfected with scrambled siRNA and AKT siRNA (50 and 100nM). After 48 h, cells were lysed and proteins were immunoblotted with antibodies against p-Akt, Akt, XIAP, and ß-actin. **(D)** Gene silencing using XIAP siRNA inactivates AKT in K562 cell line. K562 cells were transfected with scrambled siRNA and XIAP siRNA (50 and 100nM). After 48 h, cells were lysed and proteins were immunoblotted with antibodies against XIAP, p-Akt, Akt,and ß-actin.

Activation of AKT is involved in regulation of XIAP expression in many cancers including hematological malignancies [14, 40]. We and other have shown significant association of XIAP and AKT in clinical samples [4, 5, 17]. AKT and XIAP are functionally associated with each other through a feedback mechanism in various cancer cells [4, 5, 41]. We therefore sought to determine the functional link of AKT and XIAP in leukemic cells. K562 and U937 cells were treated with embelin and immunoblotted with p-AKT and XIAP antibodies. As shown in Fig 6A and 6B, AKT is constitutively activated in untreated K562 and U937 cells as determined by the status of phosphorylated AKT at Ser 473. Interestingly, embelin treatment dephosphorylates AKT and downregulates XIAP expression implicating a link between XIAP and AKT. To check for the action of embelin on other Inhibitor of Apoptosis proteins such as cIAP1 and cIAP2, we treated leukemic cells K562 and U937 with increasing doses of embelin. As shown (Fig 6A and 6B, S2A and S2B Fig), treatment of leukemic cells with embelin caused down regulation of XIAP at 25 and 50 uM. Densitometric analysis showed that embelin induced downregulation of cIAP1 expression only at higher concentration in K562 cell line and with little or no effect observed in U937 cells. Similarly no appreciable changes were observed in cIAP2 expression levels in both these cell lines. Collectively these data suggests that embelin predominantly targets PI3-kinase/Akt and XIAP pathway. This was further confirmed by using gene silencing approach with specific small interfering RNA (siRNA) of AKT and XIAP. We first transfected K562 cell line with AKT specific siRNA and level of p-AKT and XIAP were determined by immunoblotting against various antibodies. As shown in Fig 6C, gene silencing of AKT caused inactivation of p-AKT and reduction of XIAP expression in K562 cells. Similarly, knockdown of XIAP expression in K562 dephosphorylates AKT (Fig 6D). These data clearly demonstrated that expression of XIAP and activated AKT are functionally associated in leukemic cells to promote their survival and growth.

### Combination of XIAP and PI3-kinase inhibitor potentiates apoptotic cell death in leukemic cells

**Fig 7:**
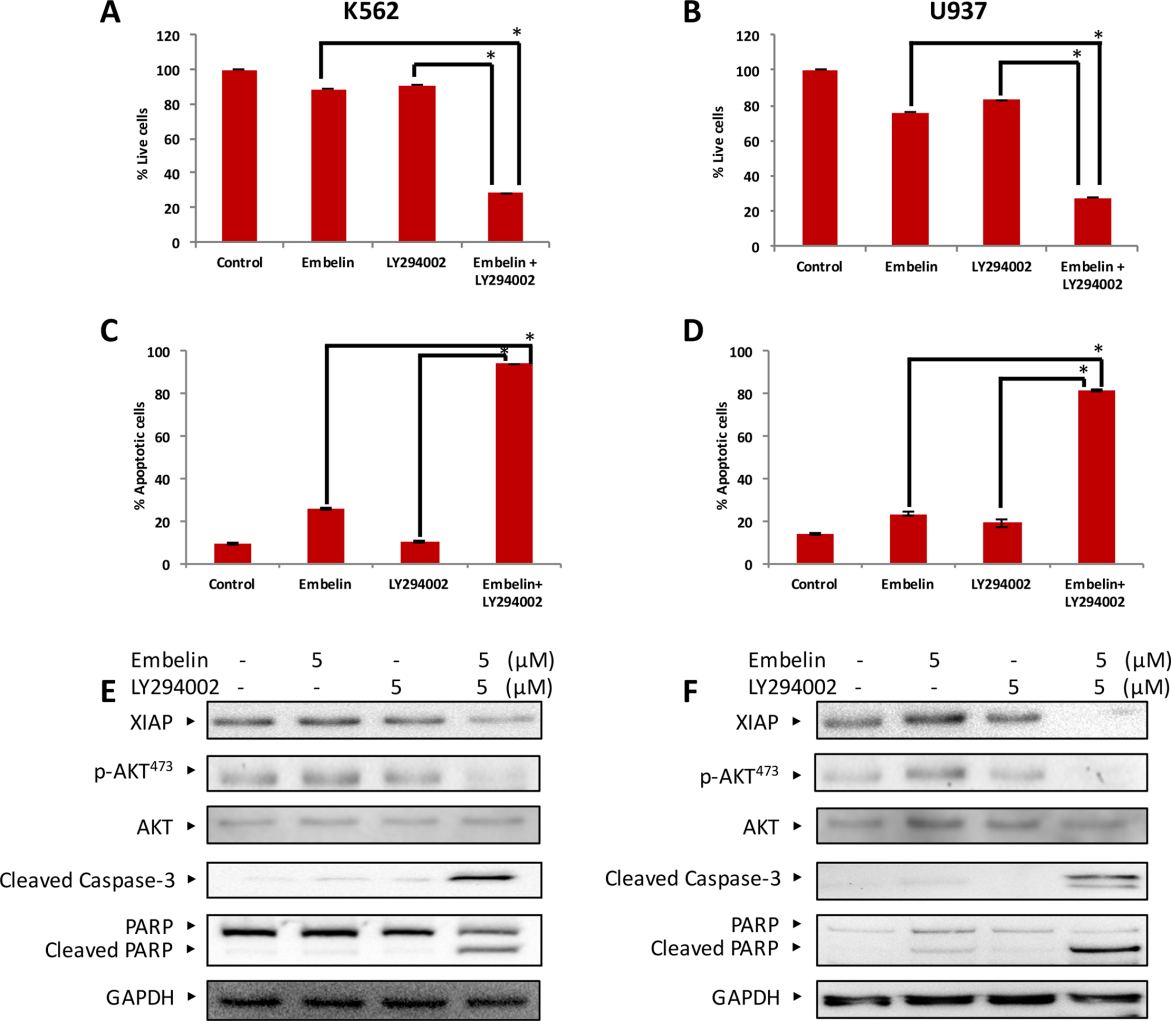
Co-treatment of leukemic cell lines with embelin and LY294002 potentiated apoptosis. K562 **(A)** and U937 **(B)** cells were treated either with 5 μM embelin or 5 μM LY294002 alone or in combination for 24 h and cell proliferation assays were performed using MTT as described in Materials and Methods. The graph displays the mean +/- SD of three independent experiments for all the doses. *P < 0.001. Subtoxic doses of Embelin and LY294002 potentiated apoptosis in leukemic cells. K562 **(C)** and U937 **(D)** cells were treated either 5 μM embelin or 5 μM LY294002 alone or in combination for 24 h, cells were subsequently stained with fluorescein-conjugated annexin-V antibody and propidium iodide (PI) and apoptosis was analyzed by flow cytometry. Percentage of apoptotic cells (early + late apoptosis) were calculated. The graph displays the mean +/- SD (standard deviation) of three independent experiments. Subtoxic doses of embelin and LY294002 potentiated the activation caspases in leukemic cells. K562 **(E)** and U937 **(F)** cells were treated either with 5 μM embelin or 5 μM LY294002 alone or in combination for 24 h. Cells were lysed and equal amount of proteins were immunoblotted with antibodies against XIAP, p-Akt, Akt, cleaved caspase-3 and PARP. GAPDH was used to measure equal loading.

Our data showed a link between XIAP and AKT, suggesting that targeting of XIAP and AKT together may augment apoptotic cell death in leukemic cells. Therefore, we used an approach of introducing sub-toxic dose of embelin (5 μM) and LY294002, a PI3-kinase inhibitor (5μM), alone and in combination on leukemic cells. These data demonstrates that combination of PI3-kinase inhibitor LY294002 along with embelin significantly inhibits cell viability by inducing caspase-mediated apoptosis (Fig 7A–7D). Western blot data demonstrated that co-treatment of embelin with LY294002 in leukemic cell lines caused downregulation of XIAP along with dephosphorylation of p-Akt, but without affecting expression of Akt, followed by activation of caspase-3 and cleavage of PARP (Figs 7E and 6F). Collectively, this data proposes that the combinational effect of embelin with LY294002 is mediated through inactivation of PI3-Kinase/Akt, thereby inducing caspase mediated apoptosis. To further confirm above results, we chose a specific Akt inhibitor (5-(2-Benzothiazolyl)-3-ethyl-2-[2-(methylphenylamino)ethenyl]-1-phenyl-1H-benzimidazolium iodide). MTT assay was performed using K562 and U937 cells using 0, 0.5, 1, 5, 10, 25 (μM) doses of the inhibitor for 24 h. Akt inhibitor caused dose-dependent inhibition of cell proliferation (S3A and S3B Fig). Next, we tried to examine potential synergy between Akt inhibitor at low dose (1 μM) and embelin (5 μM) at sub-toxic dose. Embelin was able to synergise its effect with Akt inhibitor as seen in S3C and S3D Fig.

## Discussion

Deregulation of programmed cell death, i.e., apoptosis, and related signaling pathways are involved in growth and proliferation of malignant cells and are critical processes in sustaining the unlimited progression and survival in cancer cells. These pathways are viable targets for designing effective drugs for the management and prevention of cancer. Many studies have demonstrated that XIAP is a potential therapeutic target for cancer cells as overexpression of XIAP is found in many cancers [42–44] and has been linked to the resistance to many therapeutic agents [45]. Interestingly, gene silencing using siRNA technology restores chemo-sensitivity of drugs to various cancer cell lines [46, 47].

Our data has shown that embelin causes a dose-dependent apoptotic cell death in leukemic cells via inducing apoptosis, supporting previous findings that small molecular inhibitors such as embelin can be used to inhibit XIAP in a variety of cancers including the induction of apoptosis [4, 5, 48]. Embelin showed to mediate apoptosis along with significant increase in DNA strand breaks, providing evidence that embelin can induce cell death, specifically in leukemic cell lines. These data also showed that embelin-induces dose-dependent loss of MMP and accumulation of cytochrome *c* into the cytosol in leukemic cells. These findings remain promising as mitochondria are important cell organelles that are key players in the apoptotic process [49]. Activation and cleavage of PARP are also important processes for inducing DNA breakage and cell death. In this study embelin, induced the activation of caspase-9, sequential activation of caspase-3 and PARP, and resulted in execution of intrinsic apoptosis in leukemic cells. Furthermore, our data also showed that co-treatment of K562 and U937 cells with low doses of embelin and LY294002 synergistically induced antiproliferative effect and caspase activation.

PI3-kinase mediated AKT activity is found in mitochondrial matrix as well as in its membranes [50] and is known to protect the integrity of mitochondrial membrane involving hexokinases [51]. Alteration in expression levels of BCL2 proteins and inhibition of PI3-Kinase/Akt pathway have been found to be the underlying cause of loss of MMP [52]. Our results are in concordance with previous findings. Activated AKT has been found to be functionally associated with XIAP expression in many cancers. We have previously shown the presence of a feedback loop association between AKT and XIAP in the pathogenesis in many cancers [4] and that co-targeting of XIAP and AKT pathway augmented apoptotic cell death in cancer cells [4, 5]. Overall, this study has provided support and further evidence that embelin mediated inhibition of AKT activity and inhibition of XIAP expression suggests a vital link between XIAP and AKT, and more importantly that targeting of XIAP and AKT together could potentially augment apoptotic cell death in leukemic patients.

## Conclusion

In summary, embelin-induces inhibition of cell proliferation of leukemic cells via inducing intrinsic apoptotic pathway. Furthermore, targeting XIAP and AKT simultaneously induced efficient apoptotic cell death in leukemic cells. These findings suggest that XIAP expression is an important molecular target for therapeutic intervention of such malignancies.

## Supporting information

S1 Fig Embelin-induced autophagy in leukemic cell lines. K562 **(A)** and U937 **(B)** cells were incubated with embelin at the indicated concentrations for 24 h. Total cell lysates were resolved by SDS-PAGE and immunoblotted with LC3 or GAPDH.
(TIF)

S2 Fig Effect of embelin on cIAP1 expression in leukemic cell lines. The cIAP1 bands from Fig 6A and 6B were measured using densitometric analysis and normalized with GAPDH.
(TIF)

S3 Fig Co-treatment of leukemic cell lines with embelin and Akt inhibitor 5-(2-Benzothiazolyl)-3-ethyl-2-[2-(methylphenylamino)ethenyl]-1-phenyl-1H-benzimidazolium iodide) induced apoptosis. K562 **(A)** and U937 **(B)** cells were treated with different concenterations of Akt inhibitor and incubated for 24 h. Viability was determined by using MTT assay as mentioned in Materials and Methods. K562 **(C)** and U937 **(D)** cells were treated either with 5 μM embelin or 1 μM Akt inhibitor alone or in combination for 24 h and cell proliferation assays were performed using MTT as described in Materials and Methods. The graph displays the mean +/- SD of three independent experiments. *P<0.001.
(TIF)
