# Supplementary figures and images for: Targeting of X-linked inhibitor of apoptosis protein and PI3-kinase/AKT signaling by embelin suppresses growth of leukemic cells
Source: PLoS One. 2017 Jul 13;12(7):e0180895. doi: 10.1371/journal.pone.0180895 (PMC5509148; doi:10.1371/journal.pone.0180895)

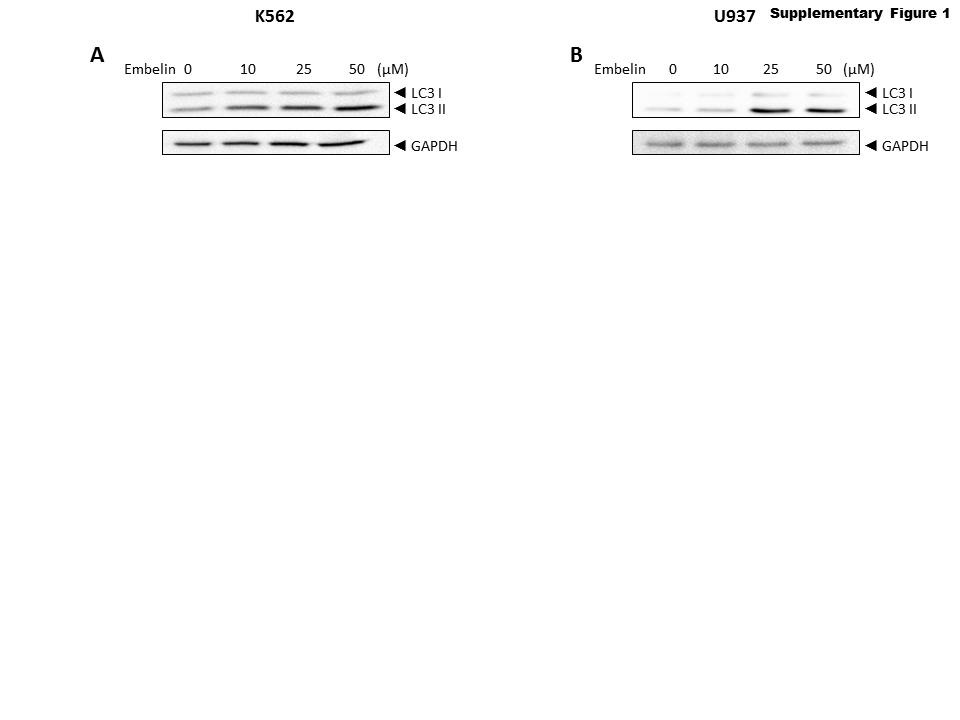

Supplement: S1 Fig — K562 (A) and U937 (B) cells were incubated with embelin at the indicated concentrations for 24 h. Total cell lysates were resolved by SDS-PAGE and immunoblotted with LC3 or GAPDH. (TIF) [file pone.0180895.s001.tif]

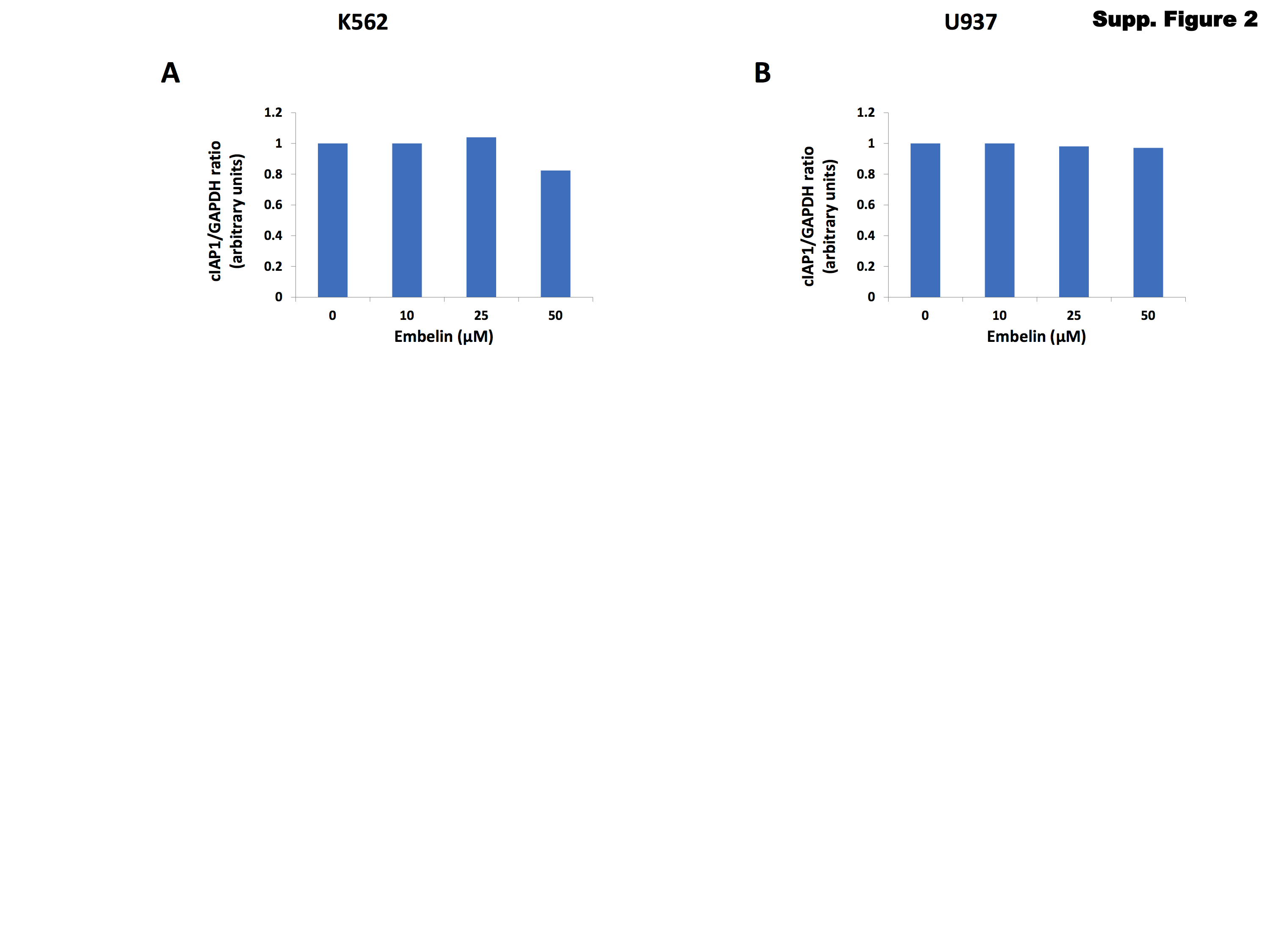

Supplement: S2 Fig — The cIAP1 bands from Fig 6A and 6B were measured using densitometric analysis and normalized with GAPDH. (TIF) [file pone.0180895.s002.tif]

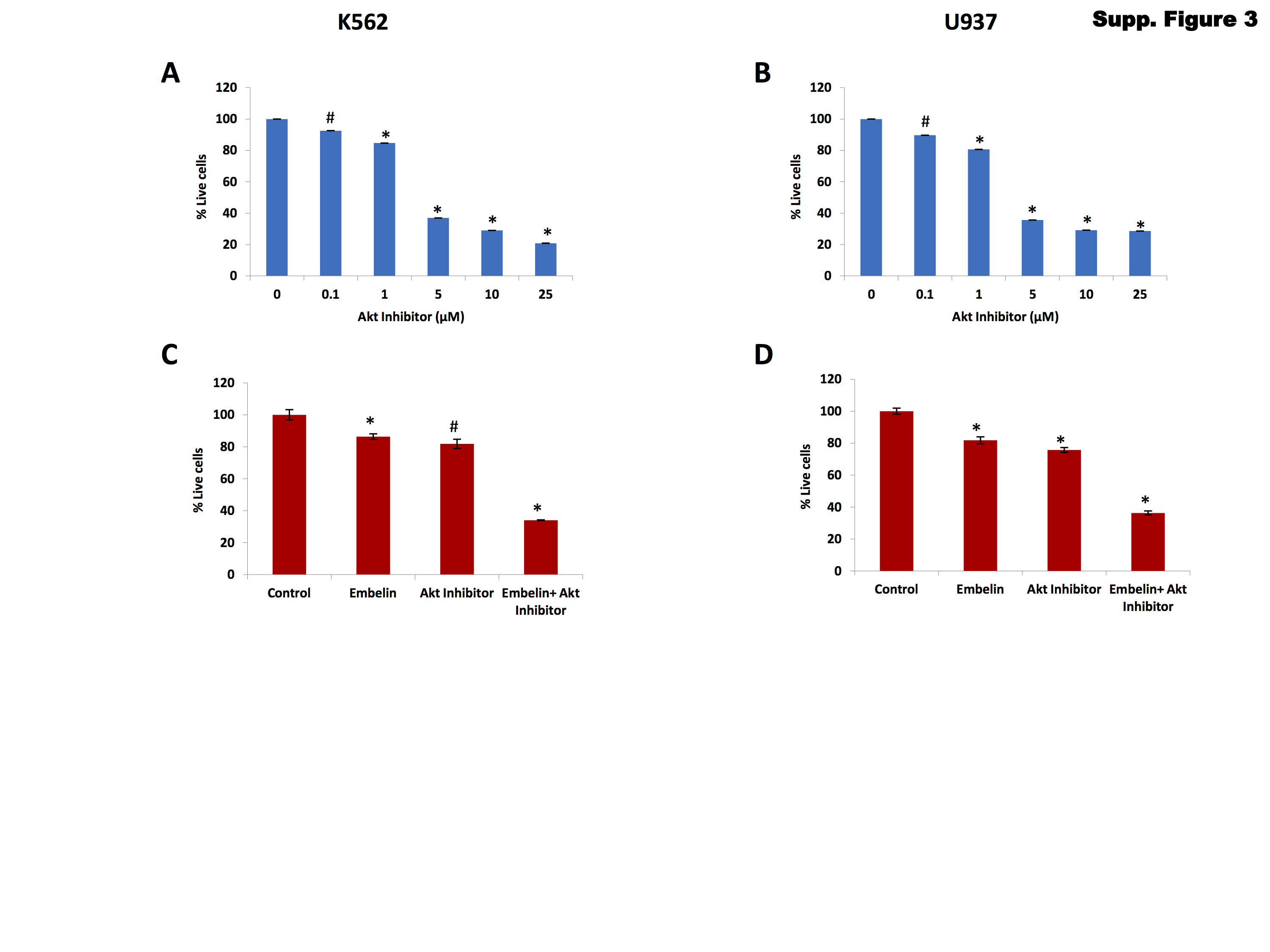

Supplement: S3 Fig — K562 (A) and U937 (B) cells were treated with different concenterations of Akt inhibitor and incubated for 24 h. Viability was determined by using MTT assay as mentioned in Materials and Methods. K562 (C) and U937 (D) cells were treated either with 5 μM embelin or 1 μM Akt inhibitor alone or in combination for 24 h and cell proliferation assays were performed using MTT as described in Materials and Methods. The graph displays the mean +/- SD of three independent experiments. *P<0.001. (TIF) [file pone.0180895.s003.tif]
